# MonoMAC syndrome with GATA2 novel mutation: A case report

**DOI:** 10.1016/j.lrr.2022.100346

**Published:** 2022-08-29

**Authors:** Petra Belohlavkova, Katerina Hrochova, Ilona Fatorova, Pavel Zak

**Affiliations:** a 4th Department of Internal Medicine – Haematology, Charles University Hospital Hradec Kralove, Czech Republic; b Institute of Clinical Biochemistry and Diagnostics, Charles University Hospital Hradec Kralove, Czech Republic

**Keywords:** GATA2 deficiency, MonoMAC syndrome, Myelodysplastic syndrome

## Abstract

GATA2 deficiency was first identified in 2011 and have been reported over 500 individuals with GATA2 mutations. The onset of symptoms ranges from early childhood to late adulthood but very often the diagnosis is made between adolescence and early adulthood. These patients can be relatively asymptomatic or have life threatening diseaseas (myelodysplastic syndrome, acute leukemia). We describe case of 30-years old women with GATA2 novel mutation who present by primary lymphedema, myelodysplastic changes in bone marrow, monocytopenia and history of several recurrent infections (bacterial, mycobacterial). The case illustrates the diagnostic difficulties in identifying GATA2 deficiencies.

## Highlights

• GATA2 deficiency is a grouping of several disorders caused by common defect in GATA2 genes.
• Age at disease onset ranges from early childhood to late adulthood, and clinical presentations range from asymptomatic to life-threatening infections, leukemia, and respiratory failure.
• GATA2 mutations were identified as a significant MDS/AML genetic predisposition.

## 1 Introduction

The GATA2 gene is located on chromosome 3q21.2 and encodes a zinc finger transcription factor. GATA2 is a key transcriptional regulator of hematopoiesis, crucially involved in hematopoietic stem cell activity and self-renewal, influences myeloid and erythroid progenitor cell differentiations or erythroid precursor cell maintenance [1, 2]. GATA2 deficiency leads to the disorder with pleiotropic clinical manifestation including myelodysplastic syndrome (MDS) and acute leukemia (AML), immunodeficiencies with high frequency of viral, bacterial or fungal infections, pulmonary manifestations (aleveolar proteionosis), vascular manifestations (lymphedema) or deafness [3]. GATA2 is required for normal and complete maturation of natural killer cells (NK) and this dysfunction is fundamental for the immunological state of patients. Emberger and MonoMAC syndrome present possible clinical variants of GATA2 deficiency [4]. Emberger syndrome is characterised by primary lymphedema generally confined to the lower limbs and genitals, sensorineural hearing loss and myelodysplastic syndrome, conversely in MonoMAC syndrome we can see monocytopenia, mycobacterial infection, pulmonary alveolar proteinosis and NK a B cell deficiencies.

## 2 Case presentation

**Fig. 1:**
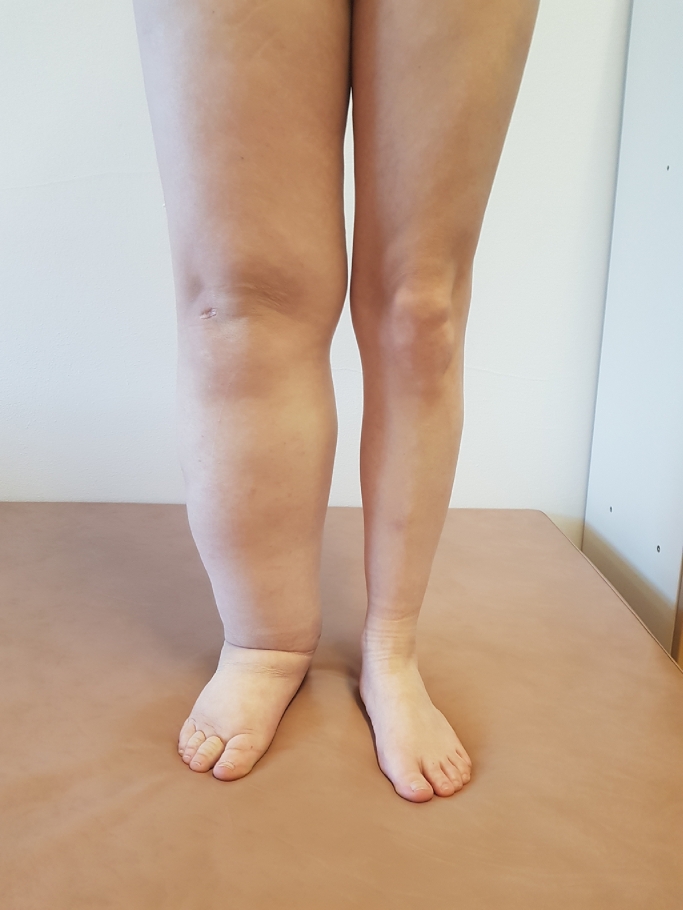
Primary lymphedema.

We describe the case of a 30-year-old woman who was referred in July 2021 to our department with suspected myelodysplastic syndrome. In the history of the patient, we found repeated skin infections (erysipelas) and pulmonary infections. The patient suffers from primary lymphedema of the right lower limb (Fig 1) since childhood. Her parents have no health problems and her 5-year-old daughter is healthy too. In June 2020 she was treated for pulmonary infection and an infectious agent has been proven Mycobacterium avium. Patient was treated with anti-infectious therapy (ciprofloxacin, ethambutol, rifampicin) six months. In March 2021, she suffered from COVID-19 pneumonia and hospitalization was necessary. Oxygen therapy was needed and the patient was treated with remdesivir, corticosteroids and antibiotics (clarithromycin).

**Fig. 2:**
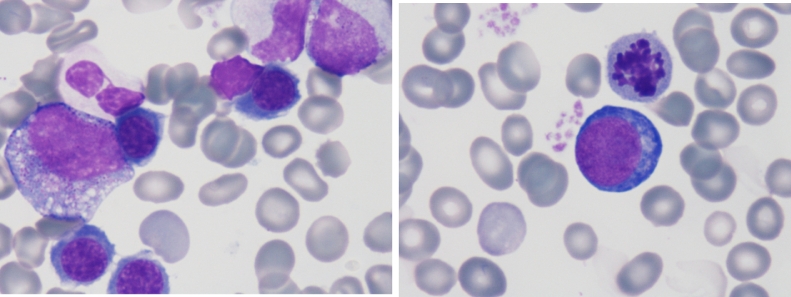
a, b: Bone marrow findings.

Blood count of the patient reveald leukocytopenia with white cells count 1.5 × 10^9^/l, 76% neutrophils, 4% rods, 15% lymphocytes, 6% basophils and 0% monocytes. The value of hemoglobin and platelets was normal. The marrow finding showed dysplastic changes in the red and white rows with normal blast count (MDS with refractory cytopenia with multlineage dysplasia) (Fig 2a,b). Cytogenetic examination confirmed normal karyotype. Next-generation sequencing (NGS) was performed and was identified GATA2 deficiency c.354dup, p.(Ser119GlufsTer66) in the second zinc finger domain, BCOR mutation c.2607T>*A*, p.(Tyr869Ter) and ATM mutation c.1229T>C, p.(Val410Ala).

**Fig. 3:**
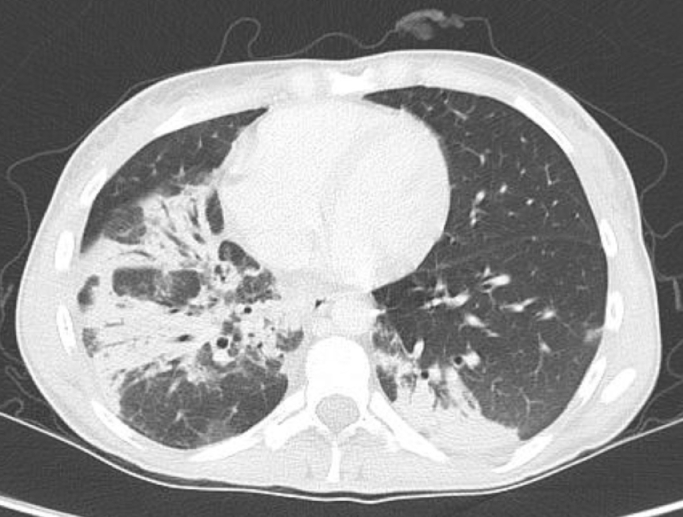
HRCT scan.

After a previous pulmonary infection patient complained of cough and shortness of breath and a pulmonary examination including high-resolution computed tomography (HRCT) of lung was added. HRCT scan showed pulmonary emphysema, lung interstitium involvement and central bronchiectasia (Fig 3). The patient has started bronchodilator therapy (tiotropium/olodaterolum, ipratropium) and breathing problems have been improving. Currently, the patient is regularly monitored and she is treated supportively (administration of antibiotics or immunoglobulins, lymphatic drainage). We plan to check bone marrow examination to evaluate the number of blasts or new mutations. Nevertheless, due to the high risk of disease progression is our patient certainly indicated for hematopoietic stem cells transplantation (HSCT).

## 3 Discussion

The median age of clinical onset of GATA2 deficiency is 18 years and has been proven that the severity of clinical symptoms inversely correlates with cytopenia. An analysis of a group of French and Belgian patients with GATA2 deficiency shows that the development of the primary lymphedema and infections in the first decade is typical disease presentation [5]. In the second decade infections continue and hematological changes often begin to appear. According to this study it has been found that probability of development of MDS/AML is 39% at the age 20 and 80% at the age 40. All children and young patients with cytopenia unclear causes should undergo examination of the bone marrow including molecular - genetic testing. The EWOGMDS study showed that 7% of all primary MDS in pediatric age had GATA2 deficiency and even 15% of advanced forms MDS [6]. Cytogenetic aberrations are frequently found too. The most frequent are monosomy 7 or del 7 which be described in 41% among all published studies. The second most common cytogenetic alteration is the trisomy 8, that was identified in 15% cases among published studies [7]. Nevertheless, the presence of del 5 and the complex karyotype have not been described yet.

MDS-associated mutations are common in patients with GATA2 deficiency and were indentified mutations in *SETBP1, ASXL1, RUNX1, CBL, EZH2, IDH2, STAG2, IKZF1* or *TP53* and the others. According to the literature, almost in 30% of cases were detected *ASXL* mutation and prognosis of these patients appears to be very poor [7], [8], [9]. In our patient we have found mutation BCOR that is occurred in 4–6% of MDS patients. The clinical impact of these mutation is controversial [10]. According to some authors, survival is not influenced by this mutation but some authors attribute this mutation a negative effect on survival. The second mutation in our case represents ATM mutation, which occurs very rarely in MDS patients and appears to be non-pathogenic [11].

GATA2 is probably a very important predisposing factor but secondary genetic events are required for development of hematologic malignancy. The mechanism underlying the development of MDS has not yet been fully discovered. But it is hypothesid that cytopenia and repeated infections lead to stress in bone marrow with the possibility of development MDS or AML [12].

Common manifestation of GATA2 deficiency is the infections they involve non-tuberculosis mycobacterium infections (in 53% of patients), bacterial infections (Clostridium difficile, Pneumocysta jiroveci) or aspergillosis (in 16% of patients). Viral infections may include human papillomavirus which causes warts, herpesvirus or Epstein-Barr virus. Warts are seen on extremities and genitalia in up 50% of patients. GATA2 was shown to play a crucial role in the development of lymphatic vessels and lymphatic valves and this fact leads to development of lymphedema (often arising in infancy or childhood). Patients have a higher risk of developing thrombosis that is likely multifactorial and this risk persists after HSCT [5, 6].

The main pulmonary feature of GATA2 is the development of pulmonary alveolar proteinosis (PAP). This is a rare lung disease in which a type of protein builds up in alveoli. PAP in GATA2 deficiency is due to macrophage dysfunction. Pulmonary involvement has a typical computed tomography (CT) scan picture but for definitive confirmation it is necessary to perform bronchoalveolar lavage. Some patients with PAP can develop some serious comlications as pulmonary arterial hypertension, loss of volume or diffusion or pneumonia Congenital deafnes has been observed in about 20 – 25% of patients with GATA2 and is related to the critical role of GATA2 in vestibular morphogenesis of semicircular ducts and generation of the perilymphatic space around the inner ear's semicircular canals [5, 6].

The clinical picture of our patient with GATA2 corresponds to MonoMAC syndrome that was first described in 2010 and in 2011 was linked to discovered mutations in the GATA2 gene. These patients suffer from preexisting monocytopenia, B-cell and NK-cell lymphopenia, reduction/lack of CD56 NK cells and dendritic cells, inverted ratio of CD4:CD8 cells, and chronic neutropenia [13]. The second typical feature of the syndrome is the development of non-tuberculosis mycobacterium infections. The problems with determining the correct diagnosis of MonoMAC syndrome we can explain by the diversity of clinical features and lack of medical knowledge about GATA2 deficiency.

At present, there are no clear guidelines regarding patient monitoring and care but the only curative treatment for patients with GATA2 deficiency is HSCT. The indication for HSCT is due to MDS, but also by recurrent infections, pulmonary deterioration or secondary serious organ damage. Unfortunately, the HSCT therapy includes many questions such as the optimal timing for transplantation, the choice of the best conditioning regimen, donor type and graft cell source. The EWOG-MDS 2017 guidelines on MDS-Refractory childhood cytopenia (RCC) recommend watchful waiting in the absence of high-risk cytogenic changes and stable blood counts [14]. However, it is clear that over time there will appear the progression of the disease or the development of complications. Therefore the strategy „the watch and wait “ strategy could not be safe.

Therefore the majority of patients with symptomatic GATA2 deficiency will need HSCT due to fact that the prognosis after MDS/AML diagnosis is poor. The decision to perform HSCT in the patient includes the occurrence of serious infections, progressive cytopenias, myeloid progression with cytogenetic changes or mutations and pulmonary alveolar proteinosis. Specific conditioning regimens have been selected for individuals. Non-myeloablative conditioning regimen are preferred when bone marrow is hypocellular, instead, myeloablative regimens are used when bone marrow is hypercellular with excess of blasts. The most discussed question is the optimal timing for HSCT that can prevent progression to MDS with excess of blasts, AML or CMML and the development of end-organ damage [15].

In conclusion, we present a case of MonoMAC syndrome related to GATA2 deficiency with typical symptoms. Surprisingly, we have found a novel GATA2 gene mutation and two other mutations (*BCOR, ATM*) and now our patient is awaiting for HSCT. The present case illustrates that children and young patients with lymphedema, recurrent infections (viral or mycobacterial) or bone marrow failure should undergo a bone marrow examination with NGS. We hope that the published case report brings attention to this rare immunodeficiency syndrome.

## Statement of ethics

Written informed consent was obtained from the patient for publication of this case report and any accompanying images, as per the Declaration of Helsinki.

## Funding sources

This work was supported by the PROGRES Q40/08 program and by MH CZ – DRO (UHHK, 00,179,906).

## Declaration of Competing Interest

The authors report no conflict of interest.

## References

[1] Hsu A.P., Sampaio E.P., Khan J., Calvo K.R., Lemieux J.E., Patel S.Y. (2011). Mutations in GATA2 are associated with the autosomal dominant and sporadic monocytopenia and mycobacterial infection (MonoMAC) syndrome. Blood.

[2] Bresnick E.H., Jung M.M., Katsumura K.R. (2020). Human GATA2 mutations and hematologic disease: how many paths to pathogenesis?. Blood Adv.

[3] Ostergaard P., Simpson M.A., Connell F.C., Steward C.G., Brice G., Woollard W.J. (2011). Mutations in GATA2 cause primary lymphedema associated with a predisposition to acute myeloid leukemia (Emberger syndrome). Nat Genet.

[4] Hirabayashi S., Wlodarski M.W., Kozyra E., Niemeyer C.M. (2017). Heterogeneity of GATA2-related myeloid neoplasms. Int. J. Hematol.

[5] Donadieu J., Lamant M., Fieschi C., De Fontbrune F.S., Caye A., Ouachee M.E. (2018). Natural history of GATA2 deficiency in a survey of 79 French and Belgian patients. Haematologica.

[6] Wlodarski M.W., Hirabayashi S., Pastor V., Stary J., Hasle H., Masetti R. (2016). Prevalence, clinical characteristics, and prognosis of GATA2-related myelodysplastic syndromes in children and adolescents. Blood.

[7] Homan C.C., Venugopal P., Arts P., Shahrin N.H., Feurstein S., Rawlings L. (2021). GATA2 deficiency syndrome: a decade of discovery. Hum. Mutat.

[8] McReynolds L.J., Yang Y., Yuen Wong H., Tang J., Zhang Y., Mulé M.P. (2019). MDS-associated mutations in germline GATA2 mutated patients with hematologic manifestations. Leuk. Res.

[9] McReynolds L.J., Zhang Y., Yang Y., Tang J., Mulé M., Hsu A.P. (2019). Rapid progression to AML in a patient with germline GATA2 mutation and acquired NRAS Q61K mutation. Leuk. Res. Rep.

[10] Badaat I., Mirza S., Padron E., Sallman D., Komrokji R., Song J., Hussaini M.O. (2020). Concurrent mutations in other epigenetic modulators portend better prognosis in BCOR-mutated myelodysplastic syndrome. J. Clin. Pathol.

[11] Ganguly B.B., Kadam N.N. (2016). Mutations of myelodysplastic syndromes (MDS): an update. Mutat. Res. Rev. Mutat. Res.

[12] McReynolds L.J., Calvo K.R., Holland S.M. (2018). Germline GATA2 mutation and bone marrow failure. Hematol. Oncol. Clin. North Am..

[13] Shen Y., Li Y., Li H., Liu Q., Dong H., Wang B., Ye B., Lin S., Shen Y., Wu D. (2021). Diagnosing MonoMAC syndrome in GATA2 germline mutated myelodysplastic syndrome via next-generation sequencing in a patient with refractory and complex infection: case report and literature review. Infect Drug Resist.

[14] Bortnick R., Wlodarski M., de Haas V., De Moerloose B., Dworzak M., Hasle H. (2021). Hematopoietic stem cell transplantation in children and adolescents with GATA2-related myelodysplastic syndrome. Bone Marrow Transplant.

[15] Bogaert D.J., Laureys G., Naesens L., Mazure D., De Bruyne M., Hsu A.P. (2020). GATA2 deficiency and haematopoietic stem cell transplantation: challenges for the clinical practitioner. Br. J. Haematol.

